# Biosynthesis of Functional Silver Nanoparticles Using Callus and Hairy Root Cultures of *Aristolochia manshuriensis*

**DOI:** 10.3390/jfb14090451

**Published:** 2023-09-01

**Authors:** Yulia A. Yugay, Maria R. Sorokina, Valeria P. Grigorchuk, Tatiana V. Rusapetova, Vladimir E. Silant’ev, Anna E. Egorova, Peter A. Adedibu, Olesya D. Kudinova, Elena A. Vasyutkina, Vladimir V. Ivanov, Alexander A. Karabtsov, Dmitriy V. Mashtalyar, Anton I. Degtyarenko, Olga V. Grishchenko, Vadim V. Kumeiko, Victor P. Bulgakov, Yury N. Shkryl

**Affiliations:** 1 Federal Scientific Center of the East Asia Terrestrial Biodiversity, Far Eastern Branch of Russian Academy of Sciences, Vladivostok 690022, Russia; yuya1992@mail.ru (Y.A.Y.); sorokina.96@gmail.com (M.R.S.); kera1313@mail.ru (V.P.G.); avramenko.dvo@gmail.com (T.V.R.); olesya55@list.ru (O.D.K.); levina@biosoil.ru (E.A.V.); 77sat7@gmail.com (A.I.D.); crab_ol@mail.ru (O.V.G.); bulgakov@ibss.dvo.ru (V.P.B.); 2 Institute of Life Sciences and Biomedicine, Far Eastern Federal University, Vladivostok 690922, Russia; vladimir.silantyev@gmail.com (V.E.S.); vkumeiko@yandex.ru (V.V.K.); 3 Institute of Chemistry, Far Eastern Branch of Russian Academy of Sciences, Vladivostok 690022, Russia; madiva@inbox.ru; 4 Department of Molecular Diagnostics and Epidemiology, Central Research Institute of Epidemiology, Moscow 111123, Russia; bioanna95@list.ru; 5 School of Advanced Engineering Studies “Institute of Biotechnology, Bioengineering and Food Systems”, Far Eastern Federal University, Vladivostok 690922, Russia; adeoluadedibu@gmail.com; 6 Far Eastern Geological Institute, Far Eastern Branch of the Russian Academy of Sciences, Vladivostok 690022, Russia; dom101@mail.ru (V.V.I.); karabzov@fegi.ru (A.A.K.); 7 A.V. Zhirmunsky National Scientific Center of Marine Biology, Far Eastern Branch of the Russian Academy of Sciences, Vladivostok 690041, Russia

**Keywords:** green synthesis, metal nanoparticles, *rol* genes, antibacterial, anticancer

## Abstract

This study delves into the novel utilization of *Aristolochia manshuriensis* cultured cells for extracellular silver nanoparticles (AgNPs) synthesis without the need for additional substances. The presence of elemental silver has been verified using energy-dispersive X-ray spectroscopy, while distinct surface plasmon resonance peaks were revealed by UV-Vis spectra. Transmission and scanning electron microscopy indicated that the AgNPs, ranging in size from 10 to 40 nm, exhibited a spherical morphology. Fourier-transform infrared analysis validated the abilty of *A. manshuriensis* extract components to serve as both reducing and capping agents for metal ions. In the context of cytotoxicity on embryonic fibroblast (NIH 3T3) and mouse neuroblastoma (N2A) cells, AgNPs demonstrated varying effects. Specifically, nanoparticles derived from callus cultures exhibited an IC_50_ of 2.8 µg/mL, effectively inhibiting N2A growth, whereas AgNPs sourced from hairy roots only achieved this only at concentrations of 50 µg/mL and above. Notably, all studied AgNPs’ treatment-induced cytotoxicity in fibroblast cells, yielding IC_50_ values ranging from 7.2 to 36.3 µg/mL. Furthermore, the findings unveiled the efficacy of the synthesized AgNPs against pathogenic microorganisms impacting both plants and animals, including *Agrobacterium rhizogenes*, *A. tumefaciens*, *Bacillus subtilis*, and *Escherichia coli*. These findings underscore the effectiveness of biotechnological methodologies in offering advanced and enhanced green nanotechnology alternatives for generating nanoparticles with applications in combating cancer and infectious disorders.

## 1. Introduction

Silver nanoparticles (AgNPs) are important nanomaterials with increasing prominence in the fields of nanoscience and nanotechnology. AgNPs have wide applications due to their multifaceted properties in electronics, catalysis, optical sensors, light emitters, mechanics, and single-electron transistors [1,2]. They are essentially indispensable in the field of biomedicine, where they have made plausible contributions to theranostics, drug delivery, antimicrobial materials, and anticancer therapy [1,3,4].

An array of physical and chemical methods for producing AgNPs has been devised, which includes laser ablation, gamma irradiation, electron irradiation, chemical reduction, and photochemical processes [5]. However, the majority of these approaches are relatively costly and involve hazardous procedures that might cause potential environmental and biological hazards [6,7]. Significant efforts were made in the last two decades to explore alternative methods, especially engaging biological systems (microbes and plants) to synthesize metal nanoparticles [6,8,9,10] as a replacement for chemical and physical techniques. This demand is fostered by the pressing need for sustainable and eco-friendly means of producing nanoparticles [11,12,13], with green nanotechnology proving to be the key.

The utilization of plant extracts is indeed a silver bullet for the production of AgNPs [14]. This innovative approach is not only cost-effective but also highly accessible, providing a rich variety of bioactive chemical components which could act both as reducing and possibly stabilizing agents in metal nanoparticles production [15,16]. Various plant species have been examined for their metabolic capacity to reduce silver, titanium, gold, and platinum ions [17]. Moreover, the synthesis can be performed both intracellularly and with extracts from different plant parts [18]. The biosynthesized nanoparticles in multiple studies were confirmed to exhibit antioxidant, antimicrobial, and anticancer properties, moreso, with low toxicity [19,20,21].

Plant cell cultures provide a more straightforward and environmentally friendly approach for biosynthesizing nanoparticles. Mude et al. made the earliest report that *Carica papaya* callus culture could be utilized for synthesizing AgNPs in the 60–80 nm range [22]. Other cell cultures, such as *Taxus yunnanensis*, *Hyptis suaveolens*, *Citrullus colocynthis*, *Costus speciosus*, *Sesuvium portulacastrum*, *Linum usitatissimum*, and *Nicotiana tabacum*, have also been used to obtain AgNPs [23,24,25,26,27,28]. Additionally, *Michelia champaca* [29] and *Cucurbita maxima* calli [30] have been utilized to produce silver and gold nanocrystals. Peanut callus was shown to promote extracellular and intracellular gold ion reduction [31]. Extracts from the callus cultures of *Viola canescens* were used for zinc oxide nanoparticles production [32]. The controlled conditions under which these cells are grown make them independent of environmental factors; using this technique poses no threat to biodiversity or natural resources [33,34,35]. Furthermore, cell culture enables modification of the biosynthesis of macromolecules and low molecular weight compounds responsible for ion reduction via classical approaches, media modification, elicitors, cell selection, and genetic engineering [36].

While numerous plant cell cultures have been examined for their potential to produce metal nanoparticles, only a handful of studies have explored using hairy root cultures in this regard. Compared to other cell cultures, the use of hairy root cultures is more advantageous in bionanotechnology due to their rapid growth and increased yield [37,38,39]. For example, aqueous extracts prepared from hairy root cultures of *Panax ginseng*, *Artemisia tilesii*, and *Artemisia annua* were previously used to bioreduce silver ions [40,41].

The medicinal plant *Aristolochia manshuriensis*, also known as Manchurian birthwort, commonly thrives in tropical and subtropical regions. This liana is of increasing interest to researchers, being a rich store of natural compounds that exhibit therapeutic properties [42]. Furthermore, *Aristolochia* plants have been used in the biosynthesis of AgNPs with useful properties. Thus, AgNPs produced using the extract of *Aristolochia indica* leaves may be employed for *Anopheles* mosquito control [43]. The synthesis of AgNPs with pronounced antioxidant activity using the extract of *Aristolochia bracteolate* shoots has been reported by Thanh et al. [44]. The suspension, hairy roots, and calli of *A. manshuriensis*, *Aristolochia indica*, and *Aristolochia bracteolate* have been obtained for producing valuable secondary compounds [45,46,47]. However, no evidence is available regarding the capacity of *Aristolochia* cell cultures to produce metal nanoparticles.

Therefore, this study focuses on AgNPs synthesis using extracts from *A. manshuriensis* cell lines. The reducing properties of callus and hairy roots were examined and compared. Additionally, the antibacterial and cytotoxic properties of the synthesized AgNPs were assessed. Overall, this paper presents a green approach to AgNPs synthesis and provides insights into their potential applications.

## 2. Materials and Methods

### 2.1. Plant Cell Cultures, Extract Preparation, and AgNPs Synthesis

The control untransformed callus culture (designated as A1), along with the *rolC* and *rolB* transgenic hairy root variations (referred to as AC and AB lines, respectively), had been previously derived from the stems of the *Aristolochia manshuriensis* liana, as outlined in earlier reports [46,48]. The calli were cultivated on solid W medium [49], supplemented with 6-benzylaminopurine and indole-3-acetic acid (each hormone at a concentration of 1 mg/L), within a dark environment at a temperature of 25 °C. This cultivation process involved a 30-day subculture interval. Similarly, the cultivation of AC and AB cell lines was conducted in liquid W medium, enriched with indole-3-butyric acid (at a concentration of 0.5 mg/L), maintaining the same environment as the A1 cells. To obtain extracts, dried 30-day-old cultures of *A. manshuriensis* were employed. The preparation of the extract involved grinding 1 g of plant biomass in 10 mL of sterile Milli-Q water. The resultant broth underwent centrifugation at 20,000× *g* for 20 min at 4 °C. The supernatant was subjected to filtration through a membrane with a pore size of 0.45 µm (Millipore, Bedford, MA, USA) and promptly used for the subsequent biosynthesis of AgNPs, following established protocols [28,33,40]. In brief, 1 mL of the aqueous extract was blended with 9 mL of a 1 mM silver nitrate (AgNO_3_, procured from Sigma-Aldrich, Saint Louis, MO, USA) solution. The reaction mixtures were subjected to agitation using a rotary shaker ES-20 (Biosan, Rīga, Latvia) at 150 rpm for a duration of 24 h at 25 °C, while being subjected to continuous illumination from an 11 W, 4000 K lamp. The resulting AgNPs were retrieved through centrifugation at 20,000× *g* for 20 min and subsequently dispersed in sterile Milli-Q water.

### 2.2. AgNPs Characterization

The UV-Vis spectral analysis of AgNPs was carried out using a BioSpec-nano spectrophotometer (Shimadzu, Kyoto, Japan), with a resolution of 1 nm and a pathlength of 0.2 mm. To investigate the morphology of the bioengineered AgNPs, high-resolution transmission electron microscopy (TEM) was conducted utilizing a LIBRA 200FE microscope (Carl Zeiss, Oberkochen, Germany) operating at an accelerating voltage of 200 kV. In addition, ultra-high resolution scanning electron microscopy (SEM) was conducted on a S-5500 microscope (Hitachi, Japan) with an accelerating voltage set at 2.0 kV.

To determine the hydrodynamic diameter and zeta potential, nanoparticle tracking analysis (NTA) was carried out using a NanoSight NS500 instrument (Malvern Instruments, Malvern, UK). This analysis involved capturing video clips (60–90 s each) of the Brownian motion of particles at 23 °C, employing a scattering mode (Supplementary Figure S1). The size determination was based on 10 videos for each sample. The zeta potential, indicating the surface charge, was assessed using an automated algorithm that simultaneously tracked individual particles in both scattering and electrophoresis modes (Supplementary Figure S1). The collected data were processed using NTA software Version 2.2.

The crystalline nature of the *A. manshuriensis*-derived AgNPs was examined through X-ray diffraction (XRD), utilizing a Miniflex II diffractometer (Rigaku, Tokyo, Japan). This analysis operated at a voltage of 30 kV and a current of 15 mA, employing Cu/Kα radiation, within a 2θ range spanning 3–80°.

For Fourier-transform infrared spectroscopy (FTIR), an FTIR-8400 spectrometer (Shimadzu, Kyoto, Japan) was employed. The assessment was conducted within the 450–4000 cm^−1^ range, utilizing KBr sample pellets as a medium for measurement.

### 2.3. Analysis of Secondary Metabolites in Aqueous Extracts

High-performance liquid chromatography (HPLC)-UV analyses were conducted using an Agilent Technologies 1260 Infinity II LC System (Santa Clara, CA, USA) with a variable wavelength detector and an C18 column (Zorbax, 150 mm length, 2.1 mm inner diameter, 3.5 µm particle size, Agilent Technologies, Santa Clara, CA, USA). The chromatographic parameters remained consistent with the detailed description provided previously [46].

### 2.4. Antibacterial Properties of AgNPs

The bactericidal efficacy of *A. manshuriensis*-derived AgNPs against Gram-negative bacteria such as *Escherichia coli XL1 Blue*, *Agrobacterium rhizogenes K599*, and *A. tumefaciens EHA105*, as well as Gram-positive bacterium *Bacillus subtilis*, was studied using the disk-diffusion method. Bacterial strains were cultured overnight at either 37 °C (for *E. coli*) or 28 °C (for *A. rhizogenes*, *A. tumefaciens*, and *B. subtilis*) in Luria–Bertani (LB) liquid medium devoid of antibiotics. The freshly obtained bacterial cultures were spread onto LB agar plates at a concentration of 1 × 106 CFU/mL. Subsequently, sterile paper discs laden with 5, 10, 15, or 20 µg of AgNPs were placed on the bacterial lawn, followed by a 24 h incubation at the appropriate temperature. The extent of growth inhibition was calculated by measuring the diameter of the area devoid of visible bacteria, encompassing the paper disc. Each treatment was performed in triplicate.

### 2.5. Evaluation of AgNPs’ Cytotoxicity

The cytotoxic activity of biosynthesized AgNPs was examined on two distinct cell lines: mouse embryonic fibroblast cells (NIH 3T3) and mouse neuroblastoma cells (N2A), following the established protocol [33,50]. These cell lines were cultivated in a mixed growth medium, a combination of DMEM and F12 (1:1) (Gibco, Grand Island, NY, USA), containing fetal bovine serum (10%) and 1x antibiotic–antimycotic solution (Gibco, USA). The cells were seeded onto 96-well plates and allowed to reach 80% confluence. Subsequently, AgNPs at desired concentrations (1, 5, 12.5, 25, 50, and 100 μg/mL), along with Milli-Q water as a control, were introduced. Following a 72 h incubation period, the MTT assay was performed on a Bio-Rad iMark microplate reader (San Francisco, CA, USA), consistent with previous methodologies [33,46,50].

### 2.6. Statistical Analyses

The data are presented as the mean ± standard error (SE). The statistical analyses included the application of a Student’s *t*-test for comparing independent groups. To compare multiple datasets, an analysis of variance (ANOVA) was conducted. For inter-group comparison, Fisher’s protected least significant difference (PLSD) post-hoc test was utilized. The threshold for statistical significance was defined as *p* < 0.05. Statistical assessments were performed using GraphPad Prism Version 9.5.1 (GraphPad Software, Inc., San Diego, CA, USA).

## 3. Results and Discussion

### 3.1. Synthesis of AgNPs

**Figure 1:**
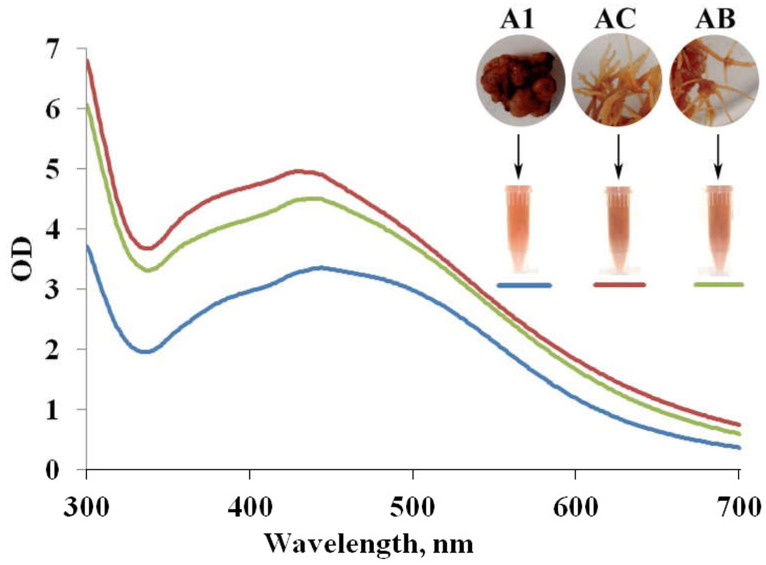
UV-Vis spectra of AgNPs obtained using aqueous extracts of the *A. manshuriensis* callus culture, *rolC*-, and *rolB*-transgenic hairy roots (A1 (blue curve), AC (red curve), and AB (green curve), respectively). The reactions were conducted for 24 h under constant light at 25 °C. The upper right section of the figure displays the visual representation of *A. manshuriensis* cell cultures and accompanying photographs showcasing the AgNPs solutions obtained through their utilization.

In this investigation, extracts derived from non-transgenic A1 callus, alongside *rolC*- and *rolB*-transgenic hairy root cultures (referred to as AC and AB, respectively) of *A. manshuriensis*, were harnessed to study the potential of *rol*-induced metabolic shifts in driving the reduction of silver ions. A discernible brown color emerged in reaction mixtures within an hour of incubation (Figure 1), indicating the possible formation of suspended AgNPs due to surface plasmon resonance (SPR) effects [51]. Markedly, the intensity of the color was more pronounced with extracts from transgenic cell cultures compared to the A1 extract (Figure 1). While the capacity of *A. manshuriensis* to synthesize AgNPs was not unexpected, due to the reducing potential of aqueous extracts from leaves and shoots of *Aristolochia* plant species [43,44,52], the constrained natural resources of the wild-growing *A. manshuriensis* liana [53] and the labor-intensive cultivation methods [54,55,56] underscored the value of hairy root cultures as an affordable and continuous source of reducing and capping agents for nanoparticle fabrication, meeting diverse industrial demands.

### 3.2. Characterization of Green-Synthesized AgNPs

The confirmation of AgNPs formation was initially carried out through UV-Vis spectroscopy. The UV absorption spectra of AgNPs synthesized within the reaction media exhibit distinct peaks at wavelengths of 444, 428, and 437 nm when using extracts from *A. manshuriensis* A1 callus, AC, and AB hairy root cultures, respectively (Figure 1). Notably, the surface plasmon resonance (SPR) bands of the resulting AgNPs displayed broadened profiles coupled with an absorption tail at extended wavelengths. This widening can be attributed to the diverse sizes and shapes of particles generated via biological processes [57]. The maximal SPR absorption values for A1, AC, and AB extracts were achieved after 24 h of incubation, measuring 3.4, 5.0, and 4.5, respectively (Figure 1). This observation indicates that hairy roots possess approximately 1.5-fold stronger reducing potential than A1 callus. This phenomenon can be attributed to the enhanced accumulation of bioactive compounds within transgenic hairy root cultures of various plant species, as previously suggested [41,58]. Notably, the *rolC* and *rolB* genes induced up-regulation of phenanthrene derivatives within *A. manshuriensis* hairy roots [46]. HPLC-UV analysis of aqueous extracts revealed that the accumulation of secondary compounds in AC and AB cell lines surpassed that of control cells by 2.3 to 3.0 times (Table 1). Consequently, the extracts sourced from *A. manshuriensis* hairy roots exhibited an augmented potential for reduction.

**Figure 2:**
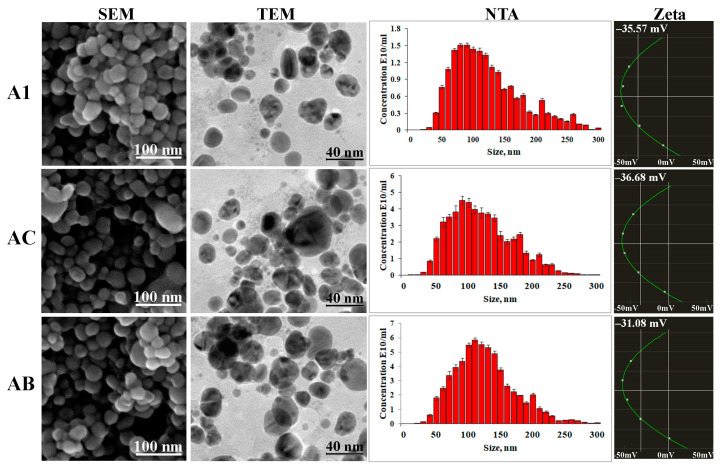
Analysis of AgNPs obtained using aqueous extracts of the *A. manshuriensis* callus culture, *rolC*-, and *rolB*-transgenic hairy roots (A1, AC, and AB, respectively). The morphology of synthesized AgNPs was evaluated using transmission and scanning electron microscopy (TEM and SEM, respectively). The particle concentration and size distribution were determined by nanoparticle tracking analysis (NTA). The electrical potential (zeta) of AgNPs was measured by a built-in automated algorithm of NTA software.

The characterization of AgNPs’ shape and size subsequent to incubation with *A. manshuriensis* extracts was executed through TEM and SEM analyses. Examination revealed the formation of particles exhibiting a spherical morphology in the size range of 10 to 40 nm (Figure 2). Prior studies have documented the production of spherical AgNPs using extracts from callus cultures of *S. portulacastrum*, *C. colocynthis*, and *Lithospermum erythrorhizon* [25,26,33].

The assessment of size distribution and concentration of *A. manshuriensis*-derived AgNPs was facilitated by nanoparticle tracking analysis (NTA) (Figure 2). It was revealed that the average hydrodynamic diameter of particles generated by A1, AC, and AB extracts was 119 ± 8 nm, 117 ± 6 nm, and 120 ± 6 nm, respectively. Correspondingly, the particle concentrations for each extract were measured at 18.32 × 10^10^, 102.10 × 10^10^, and 59.48 × 10^10^ particles/mL, respectively. Hence, there is a correlation between the absorption of the SPR bands and the estimated amount of AgNPs using NTA. Variations in AgNPs’ sizes could be attributed to discrepancies in the composition of extracts from distinct *A. manshuriensis* cell cultures, acting as capping agents. Zeta potential measurements were conducted on the green-synthesized nanoparticles to explore colloidal stability. Zeta potential values for AgNPs obtained using *A. manshuriensis* A1 callus, AC, and AB hairy root extracts were determined as −35.57 mV, −36.68 mV, and −31.08 mV, respectively (Figure 2). This implies a robust physical stability of nanosilver colloids, resistant to aggregation. These findings closely align with results from AgNPs synthesized using aqueous extracts derived from various plant species [10,13].

**Figure 3:**
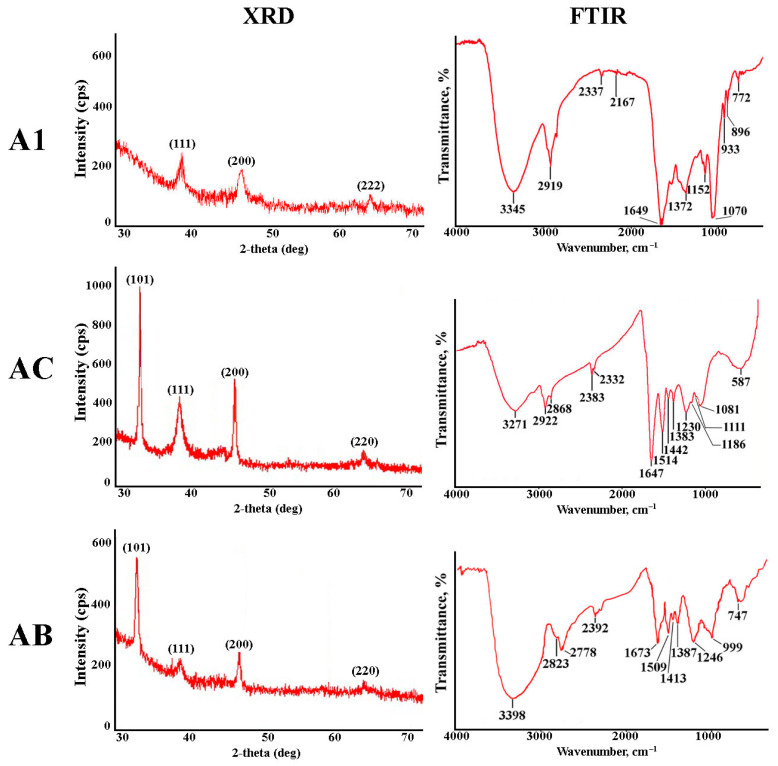
X-ray diffraction (XRD) and Fourier-transform infrared spectroscopy (FTIR) of AgNPs obtained using aqueous extracts of the *A. manshuriensis* callus culture, *rolC*-, and *rolB*-transgenic hairy roots (A1, AC, and AB, respectively).

The confirmation of the crystalline structure of the synthesized AgNPs was achieved through XRD analysis (Figure 3). The diffraction peaks noted within the 2θ range for A1, AC, and AB AgNPs corresponded to Bragg’s reflection planes (101), (111), (200), and (220) of the face-centered cubic nanostructure of metallic silver. Our findings align with the silver card No. 04-0783 in the JCPDS database. The results unequivocally prove that aspects of *A. manshuriensis* culture extracts efficiently reduced silver ions to Ag^0^ under the given reaction conditions. The XRD results affirm that the nanocrystals produced from *A. manshuriensis* are akin to previously described biologically synthesized AgNPs [59,60].

Further analysis of AgNPs was conducted using FTIR to pinpoint the biomolecules that play a fundamental role in the reduction and stabilization of silver ions (Figure 3). The stretching of O–H bonds characteristic of polysaccharides and phenolic molecules manifested in the band between 3600 and 3000 cm^−1^ [61,62]. The stretching vibrations of the C–H bond in secondary compounds, along with methyl and methylene vibrations in phospholipids or amino acids, accounted for the bands observed at 2778–2922 cm^−1^ [63,64]. The presence of primary amine (–NH_2_) groups was indicated by the band around 2332–2383 cm^−1^ [63]. Peaks at 1649 and 1514 cm^−1^ corresponded to the amide I and II bands of proteins and polypeptides. These peaks signify stretching vibrations of the C=O and N—H bonds [23]. The COO− stretching and CH_3_ bending vibrations of proteins and lipids contributed to the peak observed at 1383 cm^−1^ [65]. Absorption bands in the range of 1152–1186 cm^−1^ were attributed to hydrogen and non-hydrogen bonds of C–O stretching in polysaccharides [66]. The peak within the range of 1070–1086 cm^−1^ was linked to the stretching vibration of C–O–C and C–O bonds present in polysaccharides [50]. The bands centered around 600 and 700 cm^−1^ may arise from CH out-of-plane bending vibrations of specific *A. manshuriensis* substances linked to green-synthesized AgNPs [67]. As is evident from the findings, the reduction and stabilization of green-produced AgNPs are primarily influenced by proteins, carbohydrates, and secondary compounds within the aqueous extracts of *A. manshuriensis* cell cultures. This observation aligns with similar FTIR spectra documented for AgNPs synthesized using extracts from *A. bracteolata* shoots and *A. indica* leaves [43,44].

### 3.3. Antibacterial Activity of AgNPs

**Figure 4:**
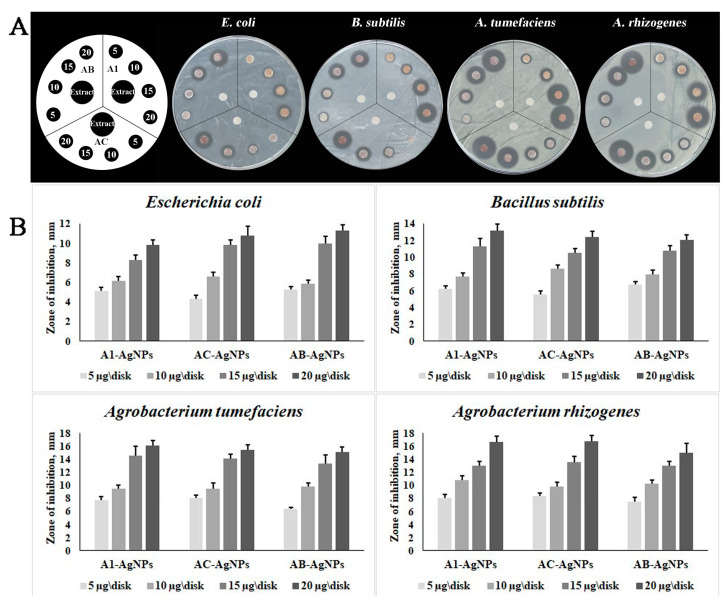
Antibacterial effect of AgNPs obtained using aqueous extracts of the *A. manshuriensis* callus culture, *rolC*-, and *rolB*-transgenic hairy roots (A1, AC, and AB, respectively) against *Agrobacterium rhizogenes*, *A. tumefaciens*, *Bacillus subtilis*, and *Escherichia coli*. (**A**), plates demonstrating increasing inhibition zones as the concentration of AgNPs rises. (**B**), graphs displaying the diameter of the inhibition zones (mm). Data are presented as the mean ± SE.

Subsequently, we delved into the investigation of the antimicrobial potential of biogenically synthesized AgNPs against bacterial strains harmful to both plants and animals, namely, *Agrobacterium rhizogenes*, *A. tumefaciens*, *Bacillus subtilis*, and *Escherichia coli*, using an agar disk-diffusion approach. Our observations unveiled a notable concentration-dependent inhibitory impact of all tested AgNPs on the viability of both Gram-negative strains, *E. coli*, *A. rhizogenes*, and *A. tumefaciens*, as well as the Gram-positive *B. subtilis* (Figure 4). Notably, the biosynthesized AgNPs exhibited a more potent inhibition of *A. tumefaciens* and *A. rhizogenes* growth compared to their effect on *E. coli* and *B. subtilis* (Figure 4). Our prior research also showcased that AgNPs derived from *LoSilA1*-transgenic tobacco calli and brown algae polysaccharides displayed a more significant bactericidal efficacy on *A. tumefaciens* as opposed to *E. coli* [28,50]. A similar trend was noted, wherein *A. tumefaciens* cells displayed heightened sensitivity to nano-Ag relative to both *E. coli* and *B. subtilis* [68]. This discovery could bear practical significance in plant biotechnology for tackling *A. tumefaciens* and *A. rhizogenes*, commonly used for genetic transformation purposes [69].

The antibacterial mechanism attributed to nanosilver encompasses a range of factors. The electrostatic interaction of AgNPs with bacterial cell walls emerges as a prominent contributor to their potent activity [70,71]. Furthermore, AgNPs are known to induce the generation of free radicals [72,73], leading to DNA damage and protein inactivation [74]. Existing evidence suggests that AgNPs can even alter membrane characteristics and permeability, thereby influencing cellular signaling [75]. Notably, the antibacterial impact of AgNPs varies between Gram-negative and Gram-positive bacteria strains, often linked to dissimilarities in the composition and structure of their cell walls [76,77]. Additionally, the variations in sizes and shapes of AgNPs exert a substantial impact on their bactericidal efficacy, as demonstrated by previous research [78,79,80,81]. However, it is imperative to highlight that, in the context of the disk-diffusion method, the passive release of silver ions predominantly underpins the toxicity of AgNPs, irrespective of their dimensions or morphology [82]. This characteristic likely holds for other materials where the free diffusion of AgNPs is constrained.

### 3.4. In Vitro Cytotoxicity Activity of AgNPs

**Figure 5:**
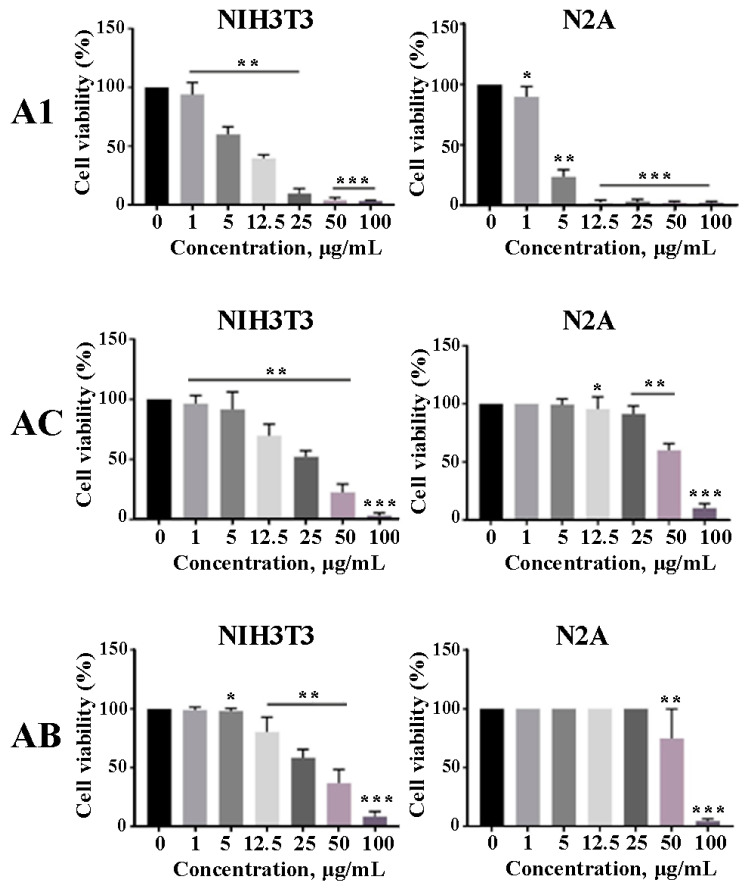
Cell viability of embryonic fibroblast (NIH 3T3) and neuroblastoma (N2A) cell lines in response to AgNPs obtained using aqueous extracts of the *A. manshuriensis* callus culture, *rolC*-, and *rolB*-transgenic hairy roots (A1, AC, and AB, respectively). Data are presented as the mean ± SE. Asterisks denote significant differences at *p* < 0.05 (*), *p* < 0.001 (**), and *p* < 0.0001 (***), Student’s *t*-test.

The colorimetric MTT test was employed to evaluate the potential cytotoxic effects of *A. manshuriensis*-synthesized AgNPs on two different cell lines: mouse neuroblastoma (N2A) and embryonic fibroblast (NIH 3T3). Figure 5 demonstrates that, in a concentration-dependent manner, the nanoparticles were effective in decreasing the viability of both N2A and NIH 3T3 cells. In particular, the growth of NIH 3T3 cells was markedly inhibited by all tested AgNPs with 50% inhibitory concentration (IC_50_) values of 7.21, 32.04, and 36.25 μg/mL for A1, A13, and A19, respectively. Moreover, AgNPs prepared using A1 calli also showed pronounced activity against N2A, with an IC_50_ value of 2.8 μg/mL. It was not possible to estimate IC_50_ values for AgNPs prepared using hairy roots extracts due to their low cytotoxicity on N2A cells. Thus, treatment of N2A with AgNPs from AC and AB cell cultures extracts did not induce cell death at the range of 1–25 μg/mL, but considerably reduced cell viability at higher concentrations (50 and 100 μg/mL). Thus, tumor cells exhibited a greater degree of viability than NIH 3T3 when treated with the same concentrations of AgNPs from hairy roots. These results suggest that despite the fact that the content of magnoflorine, an alkaloid with potential antitumor activity [83], was significantly increased in extracts from AC and AB cell cultures (Table 1), the nanoparticles synthesized from these cultures do not carry a significant amount of this anticancer compound. The comparable results were observed with AgNPs produced with *Acorus calamus* extract towards HeLa and A549 cell lines [84]. Additionally, AgNPs obtained using *Scutellaria barbata* extract were non-toxic to L929 fibroblast cells at concentrations between 2.5 and 15 g/mL [85]. On the other hand, several investigations have demonstrated that AgNPs manufactured using plant aqueous extracts possess anticancer activities [33,86]. For example, the viability of the MCF7, A549, and Hep2 cell lines was shown to diminish with increasing concentrations of *Beta vulgaris* extract-mediated AgNPs [87]. The MCF7 cell line treated with AgNPs derived from *Andrographis echioides* leaf extract yielded the same results [88]. Another study utilizing the breast cancer cell lines MCF7 and HCC70 found that when exposed to AgNPs, the proliferation index decreased significantly in both cases compared to the control [89]. Considering that the determination of the cytotoxic activities of AgNPs derived from *A. manshuriensis* was conducted alongside AgNPs generated using the extract of *Lithospermum erythrorhizon* callus culture [33], a direct comparison between these datasets becomes feasible. The data demonstrated that AgNPs synthesized from *L. erythrorhizon* exhibited nearly equivalent IC_50_ values, measuring 17 μg/mL for NIH 3T3 cells and 16 μg/mL for N2A cells. Remarkably, these values stand 2.4- and 6.4-fold greater than the corresponding IC_50_ values observed for AgNPs produced by *A. manshuriensis*, indicating a notable distinction in the cytotoxicity profiles of AgNPs synthesized from two distinct sources. Moreover, the MTT assay results on *A. manshuriensis* AgNPs indicate that the potential anticancer effects of AgNPs synthesized using aqueous extracts from medicinal plants may not necessarily represent the characteristics of alcoholic fractions or individual molecules from these plants or their cell cultures. Such contrasting outcomes highlight the intricate interplay between the nanoparticle source and resultant biological effects, necessitating further exploration for a comprehensive understanding of their implications in diverse biomedical contexts.

## 4. Conclusions

Aqueous extracts derived from *A. manshuriensis* callus cultures and hairy root lines, genetically transformed with the *rolC* and *rolB* genes from the soil bacterium *A. rhizogenes*, effectively facilitated the conversion of silver ions into colloidal nanoparticles. The synthesized AgNPs exhibited distinct surface plasmon resonance (SPR) peaks in the 428–444 nm range, displayed spherical morphology, and possessed the characteristic crystal structure associated with such particles. Examination through scanning electron microscopy (SEM) and transmission electron microscopy (TEM) unveiled particle sizes ranging from 10 to 40 nm, while the nanoparticle tracking analysis (NTA) method indicated larger sizes due to its measurement of the hydrodynamic diameter encompassing the particle. This size discrepancy is attributed to the particle’s surface properties affecting the diffusion rate within the hydrodynamic sphere. Fourier-transform infrared (FTIR) analysis affirmed the capping of particles with diverse functional groups from high and low molecular weight biomolecules of *A. manshuriensis*. The particles bore a notable negative charge, indicative of the high level of stability in resultant colloidal solutions.

Comparatively, transgenic cultures’ aqueous extracts exhibited higher quantities of phenanthrene derivatives (magnoflorine and aristolochic acids) and were 1.6 times more productive in AgNPs synthesis compared to control calli. This enhancement was corroborated by SPR peak intensities and direct NTA-based AgNPs concentration measurements. Consequently, plant genetic transformation emerges as a potential tool for engineering nanoparticle synthesis, opening avenues for tailoring particle size, shape, and distinct optical, electrical, or magnetic attributes.

Cytotoxic assessments of *A. manshuriensis*-synthesized AgNPs on mouse neuroblastoma and embryonic fibroblast cell lines underscored varying degrees of toxicity. Notably, AgNPs derived from callus cultures exhibited 2.6 times higher toxicity against tumor cells than normal cells, positioning them for further pharmacological exploration. In contrast, AgNPs from hairy root cultures displayed a limited impact on fibroblast cells and showed no neuroblastoma toxicity at doses up to 25 μg/mL. This unexpected observation suggests that the primary antitumor compound magnoflorine might not be efficiently absorbed by biosynthesized AgNPs. However, all generated AgNPs showcased comparable antimicrobial activity against pathogenic bacteria, highlighting the weak correlation between bactericidal properties and the type of cell culture. In summation, these findings underscore the potential of biotechnological strategies in green nanotechnology, offering avenues for developing metal nanoparticles with potential biomedicine and biotechnology applications.

## Figures and Tables

**Table 1:** The concentration (µg/mL) of phenanthrene derivatives in aqueous extracts from callus culture, *rolC*-, and *rolB*-transgenic hairy roots (A1, AC, and AB, respectively) of *A. manshuriensis* determined using HPLC-UV.

	A1	AC	AB
Magnoflorine	35.3 ± 4.9 ^C^	203.8 ± 24.5 ^A^	154.2 ± 15.4 ^B^
Aristolochic acids *	3.68 ± 0.46 ^B^	10.41 ± 1.28 ^A^	11.87 ± 1.39 ^A^

Data are presented as the mean ± SE. Different superscript letters specify statistically significant differences of means (*p* < 0.05) in the rows, Fisher’s PLSD test. * The sum of aristolochic acid-I, II, IIIa, and IVa/b.

## Data Availability

All data are contained within the article and Supplementary Materials. The datasets generated during and/or analyzed during the current study are available from the corresponding author upon reasonable request.

## Supplementary Materials

The following supporting information can be downloaded at: https://www.mdpi.com/article/10.3390/jfb14090451/s1, Figure S1: Representative nanoparticle tracking analysis (NTA) video frames and electrical potential (Zeta) measurements.
