# Supplementary material for: Biosynthesis of Functional Silver Nanoparticles Using Callus and Hairy Root Cultures of Aristolochia manshuriensis
Source: J Funct Biomater. 2023 Sep 1;14(9):451. doi: 10.3390/jfb14090451 (PMC10532211; doi:10.3390/jfb14090451)
Supplement: Supplementary file 1 [file jfb-14-00451-s001.zip › jfb-2542720-supplementary.pdf]

Journal of Functional Biomaterials

Supplementary Materials

Biosynthesis of Functional Silver Nanoparticles Using Callus and Hairy Root Cultures of *Aristolochia manshuriensis*

Yulia A. Yugay [1], Maria R. Sorokina [1], Valeria P. Grigorchuk [1], Tatiana V. Rusapetova [1], Vladimir E. Silant'ev [2,3], Anna E. Egorova [4], Peter A. Adedibu [5], Olesya D. Kudinova [1], Elena A. Vasyutkina [1], Vladimir V. Ivanov [6], Alexander A. Karabtsov [6], Dmitriy V. Mashtalyar [3], Anton I. Degtyarenko [1], Olga V. Grishchenko [1], Vadim V. Kumeiko [2,7], Victor P. Bulgakov [1] and Yury N. Shkryl [1,5,*]

1 Federal Scientific Center of the East Asia Terrestrial Biodiversity, Far Eastern Branch of Russian Academy of Sciences, Vladivostok 690022, Russia; yuya1992@mail.ru (Y.A.Y.); sorokina.96@gmail.com (M.R.S.); kera1313@mail.ru (V.P.G.); avramenko.dvo@gmail.com (T.V.R.); olesya55@list.ru (O.D.K.); levina@biosoil.ru (E.A.V.); 77sat7@gmail.com (A.I.D.); crab_ol@mail.ru (O.V.G.); bulgakov@ibss.dvo.ru (V.P.B.)
2 Institute of Life Sciences and Biomedicine, Far Eastern Federal University, Vladivostok 690922, Russia; vladimir.silantyev@gmail.com (V.E.S.); vkumeiko@yandex.ru (V.V.K.)
3 Institute of Chemistry, Far Eastern Branch of Russian Academy of Sciences, Vladivostok 690022, Russia; madiva@inbox.ru
4 Department of Molecular Diagnostics and Epidemiology, Central Research Institute of Epidemiology, Moscow 111123, Russia; bioanna95@list.ru
5 School of Advanced Engineering Studies "Institute of Biotechnology, Bioengineering and Food Systems", Far Eastern Federal University, Vladivostok 690922, Russia; adeoluadedibu@gmail.com
6 Far Eastern Geological Institute, Far Eastern Branch of the Russian Academy of Sciences, Vladivostok 690022, Russia; dom101@mail.ru (V.V.I.); karabzov@fegi.ru (A.A.K.)
7 A.V. Zhirmunsky National Scientific Center of Marine Biology, Far Eastern Branch of the Russian Academy of Sciences, Vladivostok 690041, Russia
* Correspondence: yn80@mail.ru; Tel.: +007-(423)-231-21-29; Fax: +007-423-2310193

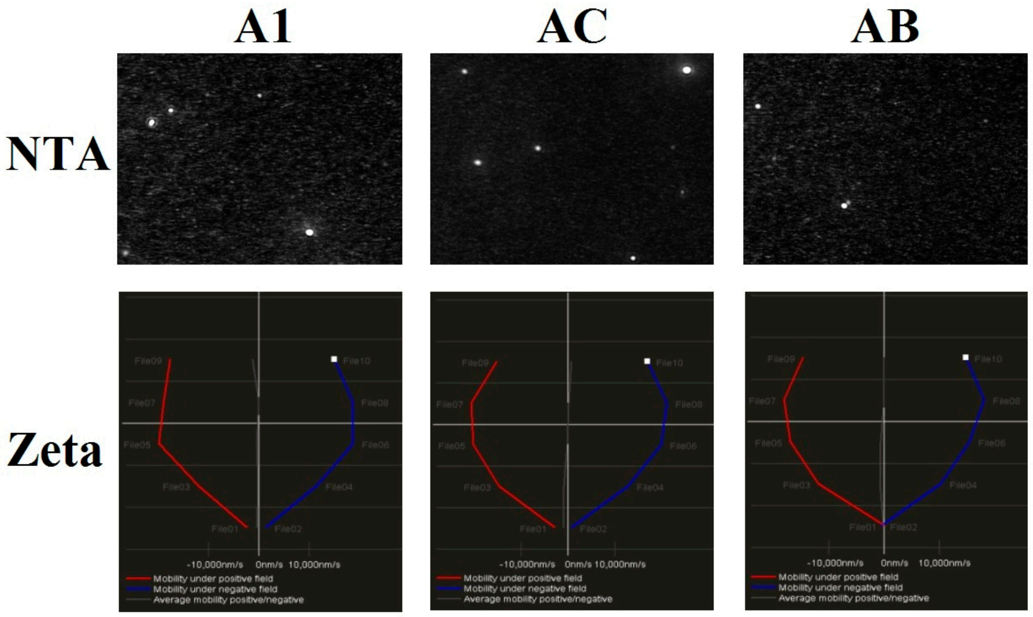

Figure S1. Representative nanoparticle tracking analysis (NTA) video frames and electrical potential (Zeta) measurements.
